# Assessment of pH-Induced Conformational Changes in Whey Protein Isolate–Dextran Conjugate Using Spectral Technology

**DOI:** 10.3390/foods14111952

**Published:** 2025-05-30

**Authors:** Qingyuan Dai, Huiqin Wang, Xiuling Zhu, Polyanna Silveira Hornung, Yuru Zhang, Wenxuan Hu, Anqi Lin, Anyi Yao, Trust Beta

**Affiliations:** 1 School of Biological and Food Engineering, Anhui Polytechnic University, Beijing Middle Road, Wuhu 241000, China; daiqingyuan9@126.com (Q.D.); 18356378352@163.com (H.W.); z2629112582@163.com (Y.Z.); 18856247276@163.com (W.H.); emusy040816@163.com (A.L.); dusharm@163.com (A.Y.); 2 Wuhu Green Food Industry Research Institute Co., Ltd., Wuwei Economic Development Zone, Wuhu 238326, China; 3 Department of Food and Human Nutritional Sciences, University of Manitoba, Winnipeg, MB R3T 2N2, Canada; polyanna.hornung@hylife.com (P.S.H.); trust.beta@umanitoba.ca (T.B.)

**Keywords:** degree of glycosylation, FTIR spectroscopy, fluorescence quenching, polarity, hydrophobicity

## Abstract

The functional properties of proteins are closely related to their structure and conformation. The effects of glycosylation and pH on the structural and conformational changes in whey protein isolate (WPI) were investigated using multispectral technology. More and higher-molecular-weight molecules of WPI–dextran conjugates (WDCs) with increased degrees of glycosylation (DGs) in SDS-PAGE occurred at the expense of band intensities of α-lactalbumin, β-lactoglobulin, and bovine serum albumin. The higher wavenumber shift in FTIR peaks of WPI after glycosylation in the Amide I, II, and III regions and the decrease in its intensity occurred. The maximum absorption wavelength (λ_max_) of UV-Vis spectra of WPI before and after glycosylation in the range of 260–290 nm showed no significant difference in a pH range of 2.0–10.0. Moreover, the UV-Vis absorption intensities of WDCs at λ_max_ around 278 nm were highly and positively correlated with their DGs. The λ_max_ and intensities of total intrinsic fluorescence spectra of Tyr and Trp residues in WDCs with an increase in DGs had an obvious redshift and decrease, respectively. Although the intensities of synchronous fluorescence spectra of individual Tyr or Trp residues in WDCs with an increase in DGs also gradually decreased, the λ_max_ of the former and latter had a blueshift and redshift, respectively. UV-Vis absorption and fluorescence spectroscopies indicated that the changes in the λ_max_ and intensity of WPI were closely related to the protonation states of carbonyl groups and free amino groups and the degree of glycosylation. This work may be beneficial for understanding the structural and conformational changes in proteins by measuring the microenvironment around Tyr and/or Trp residues in proteins using UV-Vis absorption and synchronous fluorescence spectroscopies, providing a promising technique for quantitatively monitoring the degree of glycosylation (DG) in a rapid and practical way without any chemical reagents using UV-Vis absorption spectroscopy.

## 1. Introduction

Whey protein isolate (WPI), a byproduct of cheese in the milk industry, is an ingredient widely used in the food industry due to its exceptional nutritional value, unique functional properties, and competitive cost advantage [1,2]. The excellent nutritional value of WPI originates from its high content of essential amino acids. WPI consists of β-lactoglobulin (β-Lg), α-lactalbumin (α-La), bovine serum albumin (BSA), immunoglobulin (Ig), lactoferrin (Lf), some minor enzymes, osteopontin, trefoil factors, and several growth factors [3]. β-Lg (18.3 kDa) and α-La (14.2 kDa) make up 70–80% of the total whey protein in bovine milk. The two main proteins endow WPI with excellent emulsifying, foaming, and gelation activities, which closely depend on protein solubility. However, the solubility of WPI is minimal near its isoelectric point, which impairs its functional properties [2,4].

The functional properties of proteins can be improved by physical, chemical, and enzymatic modification [5]. Although the physical approach is valued for its low cost, safety, and low loss of nutritional value, the modification effect is minimal and energy consumption is high. The chemical approach offers advantages such as quick reaction time, simple operation, and significant effects, but it also poses problems with chemical reagent residues and safety hazards. The enzymatic approach has benefits including good specificity, mild reaction conditions, and a controlled reaction rate, but it also has shortcomings such as possible bitterness after hydrolysis and fewer types of enzymes. The Maillard reaction is a nonenzymatic condensation reaction between the free amino groups of proteins, peptides, or amino acids and reducing-end carbonyl groups of poly-, oligo-, or monosaccharides. The Maillard reaction, considered a green and safe method for preparing protein–sugar conjugates, has received wide attention in recent years because only temperature, water content or humidity, and pH need to be controlled during the reaction process without the use of other chemical reagents [2]. The solubility of proteins can be significantly increased near their isoelectric point via the Maillard reaction, consequently improving their functional properties, such as stability, emulsifying, and foaming [6]. The dry heating approach of the Maillard reaction is difficult to control in terms of the extent of reaction and requires a long reaction time. Although the wet heating method of the Maillard reaction could reduce the reaction time, it is usually performed at higher reaction temperatures, which easily causes the polymerization, denaturation, and aggregation of proteins and a low degree of glycosylation (DG) [7]. A new approach to the Maillard reaction with ethanol–water pretreatment not only effectively overcomes the deficiencies of dry heating and wet heating approaches but also combines the advantages of the two classical methods and its own unique advantages, including a high reaction rate, low energy consumption, small amount of wastewater discharge, and low cost [8].

The functional properties of proteins are closely related to their structure and conformation, which are sensitive to different treatments and environmental conditions, such as glycosylation, pH, and heat [9]. The structure and conformation of proteins are altered following glycosylation through covalent bonding with sugar chains. The ionized states of peptide side chains of proteins change under different pH conditions, subsequently altering the conformation of proteins. Although mass spectrometry and nuclear magnetic resonance spectroscopy can be used to obtain the detailed chemical structure of molecules with unique benefits such as high sensitivity, selectivity, and precision, spectrum analysis is time-consuming and costly [10]. The structural and conformational changes in proteins can also be determined using rapid, sensitive, and inexpensive spectroscopic techniques such as Fourier transform infrared (FTIR), ultraviolet–visible (UV-Vis), and fluorescence spectroscopy. Different spectroscopies can capture the characteristic spectral signals of different functional groups of proteins and differ in their sensitivity. Spectroscopic techniques can be used to monitor and evaluate the interaction between electromagnetic waves and matter. FTIR spectroscopy quickly detects the vibrational properties of polarized asymmetric functional groups of molecules, which are sensitive to minute structural changes [11]. The different absorption bands in an FTIR spectrum result from different vibration frequencies of the specific chemical bonds [12]. UV-Vis spectroscopy exploits the interactions between UV-Vis light in the range from 200 nm to 780 nm and compounds. UV-Vis absorption at specific wavelengths is restricted to unsaturated groups in compounds, which are called chromophores and are responsible for their colors. Although saturated groups in compounds have no absorption in the UV-Vis region, the same saturated groups influence the absorption of chromophore groups without color change in themselves, hence the name auxochromes. Environmental conditions can change the absorption intensity and wavelength of chromophores [13]. UV-Vis spectra of proteins are mainly attributed to the peptide backbone and aromatic amino acid residues [6,14]. Fluorescence spectroscopy is a powerful technique to detect the tertiary structure (spatial structure) of proteins [15]. Fluorophores are the unsaturated components in molecules [16]. Different fluorophores can be excited after absorption of UV or visible light at specific wavelengths, subsequently emitting the fluorescence over a particular longer range of wavelengths. Fluorescence spectra of proteins only result from the fluorophores with aromatic rings, including tryptophan (Trp), tyrosine (Tyr), and phenylalanine (Phe) residues [17]. However, fluorescence spectra of proteins mainly originate from Trp and Tyr residues due to a very low quantum yield of Phe residues [18]. Additionally, fluorescence spectroscopy can characterize the changes of local microenvironmental polarity around individual Trp or Tyr residues in proteins. Therefore, different spectroscopic methods can be used to measure functional groups regardless of unsaturated and saturated groups, unsaturated groups such as peptide backbone and aromatic amino acid residues; aromatic amino acid residues in proteins are measured by FTIR, UV-Vis and fluorescence spectroscopies, respectively. In other words, FTIR, UV-Vis, and fluorescence spectroscopies can be used to evaluate the structural and conformational changes of proteins from their different functional groups. Nevertheless, there are few systematic and comprehensive studies using UV-Vis and fluorescence spectroscopy on the structural and conformational changes of natural and glycosylated WPI with varying degrees of glycosylation over a wide range of pH from 2.0 to 10.0, which would be beneficial for capturing higher sensitive and rapid spectroscopic techniques to analyze the structure and conformation of proteins that have undergone glycosylation and pH modification by monitoring specific functional groups.

The objective of the present study was to investigate the effects of glycosylation and pH on the structural and conformational changes of WPI. The structural change of WPI after covalent grafting of dextran from the Maillard reaction with ethanol–water pretreatment was confirmed using DG, SDS-PAGE, and FTIR spectroscopy. The conformational changes were determined using FTIR, UV-Vis absorption, intrinsic fluorescence emission, and synchronous fluorescence spectroscopy. The findings may be beneficial for obtaining a sensitive, rapid, and inexpensive spectroscopic technique to monitor the structural and conformational changes of proteins in different pH conditions after glycosylation. They would also provide a basis for protein modification to obtain desirable functional properties via the Maillard reaction and pH regulation.

## 2. Materials and Methods

### 2.1. Materials and Reagents

WPI was obtained from Hilmar Ingredients (Hilmar, CA, USA). β-Mercaptoethanol was acquired from Shanghai McLean Biochemical Technology Co., Ltd. (Shanghai, China). Dextran with a molecular weight of 40 kDa, glycine, lysine, methanol, *o*-phthaldialdehyde (OPA), sodium dodecyl sulfate (SDS), sodium tetraborate, disodium hydrogen phosphate, sodium dihydrogen phosphate, potassium bromide, hydrochloric acid, sodium hydroxide, Coomassie Brilliant Blue R-250, and anhydrous ethanol were supplied by Sinopharm Chemical Reagent Co., Ltd. (Shanghai, China). Deionized water was used as the aqueous solvent.

### 2.2. Preparation of WPI–Dextran Conjugates

Our previous research reported that the different DGs of WPI–dextran conjugates (WDCs) were successively prepared from five single-factor experiments, including temperature, time, weight ratio of WPI to dextran, ethanol volume fraction, and ratio of solid to liquid. The DGs of WDCs with approximate arithmetic progression were obtained for different reaction times (0.5–18 h). To explore the spectral properties of WDCs with different DGs in present paper, WDCs were prepared based on our previous experimental conditions: temperature 70 °C, weight ratio of WPI to dextran 1:3, ethanol 90% (*v*/*v*), ratio of solid to liquid 10 g/100 mL (*w*/*v*), time (0.5–18 h) [8]. Briefly, WPI (0.5 g) was added to 2 mL of deionized water at pH 7.0 and magnetically stirred for 15 min. Dextran (1.5 g) was added and then stirred for 15 min to achieve complete uniform mixing. Finally, anhydrous ethanol was added, and adjustments made to obtain 90% volume fraction of the total volume of water and ethanol solution at a solid to liquid ratio of 10% (*w*/*v*). The mixture was stirred thoroughly and then reacted at 70 °C in a water bath for 0, 0.5, 1, 3, 12, and 18 h. The reaction was terminated by cooling immediately to room temperature in an ice water bath. After the supernatants were separated, the precipitates were placed in Petri dishes, naturally dried at room temperature (25 °C) for 24 h to obtain WDCs, and then ground into powder. The powdered samples were stored at room temperature and used for further experiments. WDCs at different reaction times were recorded as WDM (0 h), WDC (0.5 h), WDC (1 h), WDC (3 h), WDC (12 h), and WDC (18 h), respectively.

### 2.3. Degree of Glycosylation

The degree of glycosylation (DG) was measured using the *o*-phthaldialdehyde (OPA) method, as described previously [8]. Briefly, 80 mg of OPA was dissolved in 2 mL of methanol, and then 5 mL of 20% (*w*/*w*) SDS, 50 mL of 0.1 mol/L sodium tetraborate, and 200 μL of β-mercaptoethanol were added. The mixed solution was diluted to 100 mL using deionized water. The final mixture was designated as the fresh OPA reagent. The protein solution (200 μL of 2 mg/mL) was mixed with 4 mL of fresh OPA reagent and then incubated in a 35 °C water bath for 2 min. Afterwards, the absorbance of the incubation solution was determined at 340 nm using a UV-Vis spectrophotometer (UV-5800, Metash, Shanghai, China). The OPA assay is not interfered with by dextran because there is no chemical reaction between dextran and OPA reagent. A solution containing 200 μL of deionized water and 4 mL of fresh OPA reagent was used as a blank control. A plot between absorbance and molar concentration of free amino acid residues was obtained using lysine as the standard curve (A = 1.4017C + 0.0096, R^2^ = 0.9996).

The DG of the glycosylated protein was calculated according to the following Equation (1):

(1) $$\mathrm{DG}\;(\%)=\frac{C_{0}-C_{t}}{C_{0}}\times 100$$

where C_0_ and C_t_ are the free amino group contents (mmol/L) in sample before and after the Maillard reaction, respectively.

### 2.4. Electrophoresis

Sodium dodecyl sulfate-polyacrylamide gel electrophoresis (SDS-PAGE) analysis was conducted according to the method described by Dai [8]. Initially, 6 μL of each sample containing 2 mg/mL protein was mixed with 24 μL of loading buffer containing 50 mM pH 6.8 Tris-Cl, 10% glycerol, 2% SDS, 2% β-mercaptoethanol, and 0.1% bromophenol blue. The mixed solution was then sealed and heated at 100 °C for 3 min. All samples were treated in the same way. Subsequently, 15 μL of each sample and a molecular weight marker were added to each well in the SDS-PAGE gel, which consisted of a 12% separating gel and a 5% stacking gel. Electrophoresis was carried out at 70 V in the stacking gel and then at 120 V in the separating gel until the dye front reached the bottom of the separating gel. The gel was then stained for up to 60 min using Coomassie Brilliant Blue R-250, followed by destaining using 10% acetic acid (*v*/*v*) containing 10% methanol (*v*/*v*) until the gel was colorless. Finally, the gel was captured using a gel documentation system to obtain a clear and bright electrophoretic map.

### 2.5. FTIR Spectroscopy

A total of 1 mg of dried sample powder and 100 mg of anhydrous KBr were mixed in an agate mortar and thoroughly ground before pressing into a thin pellet [19]. Measurements were performed at ambient temperature (25 °C) using an FTIR spectrometer (IRPrestige-21, Shimadzu Corporation, Tokyo, Japan) with a resolution of 4 cm^−1^ in a scanning wavenumber range of 500–4000 cm^−1^. Sixteen scans were performed to obtain a reasonable signal-to-noise ratio. Anhydrous KBr pellet was used as a blank control.

### 2.6. UV-Vis Absorption Spectroscopy

The absorbance of each sample solution was measured in the range of 190–500 nm at room temperature (25 °C) using a UV-Vis spectrophotometer (UV-5800, Metash, Shanghai, China) according to a previous method with slight modifications [20]. The protein concentration was chosen at 1 mg/mL to obtain appropriate absorbances such as 0.5–1.0, in the range of 260–290 nm, according to previous research [6,21,22]. The samples were dissolved in deionized water to prepare completely dissolved solutions with an initial protein concentration of 2 mg/mL and then diluted to a final 1 mg/mL with 0.01 mol/L buffer solutions of different pH values, including disodium hydrogen phosphate-citric acid (pH 2.0–6.0), phosphate buffer saline (pH 7.0–8.0), and glycine-sodium hydroxide (pH 9.0–10.0), respectively. Different preset pH values were confirmed and adjusted using 0.1 mol/L HCl or NaOH solutions. The absorption spectra were recorded in a quartz cuvette with a 1 cm light path. The corresponding buffer solution was used as a blank control.

### 2.7. Intrinsic Fluorescence Emission Spectroscopy

The fluorescence properties of sample solutions were measured according to the reported method with some modifications [23]. The sample solutions with a protein concentration of 2 mg/mL in deionized water were diluted to 0.2 mg/mL, as above for UV-Vis spectroscopy using 0.01 mol/L buffer solutions of different pH (2.0–10.0). Different preset pH values were confirmed and adjusted using 0.1 mol/L HCl or NaOH solutions. The intrinsic fluorescence emission spectra of the sample solutions were recorded at room temperature (25 °C) using a fluorescence spectrophotometer (F-7100, Hitachi Co., Ltd., Tokyo, Japan) with a scanning speed of 1200 nm/min at a voltage of 600 V. The emission spectra were collected from 290 nm to 450 nm at an excitation wavelength of 280 nm both with a slit width of 5 nm. The corresponding buffer solution without WPI was used as a control to correct for the fluorescence background.

### 2.8. Synchronous Fluorescence Spectroscopy

Synchronous fluorescence spectra were determined at room temperature (25 °C) using a fluorescence spectrophotometer (F-7100, Hitachi Co., Ltd., Tokyo, Japan) according to a previous method with slight modifications [24]. The synchronous fluorescence spectra of the same sample solutions as in the intrinsic fluorescence spectroscopic experiments were recorded from 240 nm to 360 nm at fixed 15 nm and 60 nm intervals (Δλ) between the emission wavelength and excitation wavelength both with a slit width of 5 nm. The corresponding buffer solution without WPI was used as a blank control. To obtain the net fluorescence intensity of each protein sample, the fluorescence intensity of each sample blank was subtracted from that of the corresponding sample.

### 2.9. Statistical Analysis

All experiments were conducted in triplicate, and all data were presented as mean ± standard deviation (SD). SPSS software (version 26.0, SPSS Inc., Chicago, IL, USA) was used for statistical analysis. Significant differences in the data were obtained using one-way analysis of variance (ANOVA) and Duncan’s multiple range tests with a significance level of *p* < 0.05 [25]. Different letters indicate significant differences (*p* < 0.05). Charts were plotted using Origin software (version 9.0, OriginLab Co. Ltd., Northampton, MA, USA).

## 3. Results and Discussion

### 3.1. Degree of Glycosylation Analysis

The condensation reaction between the free amino groups of protein molecules and the reducing-end carbonyl groups of sugar molecules occurs during the Maillard reaction process. The content of the free amino groups in protein will decrease with an increase in the DG. Therefore, DG is commonly used to evaluate the extent of the Maillard reaction [26].

The degrees of glycosylation (DGs) at different reaction times are shown in Table 1. The DGs of WDCs continuously increased with an extension of time. The DGs rapidly increased when the reaction time was in the range of 0.5–3 h, which was attributed to the higher content of reactive groups both in WPI and dextran at an early reaction stage, and therefore, an increase in the probability of the reaction. However, the DGs slowly increased after a reaction time of 3 h. This was due to several factors. Firstly, the decrease in the content of reactive groups both in WPI and dextran in the intermediate and final stages of reaction reduced the chances of collisions among reactive moieties. Secondly, the steric hindrance from the macromolecular chains of Maillard reaction products, such as Schiff bases and Amadori compounds, especially the long side-chain dextran of covalent grafting in WDCs, could reduce the subsequent efficiency of the Maillard reaction. Thirdly, the lower molecular flexibility of WDCs with a higher molecular weight might also reduce the reaction probability. Fourthly, the conformational change of WPI induced after covalently grafting with dextran shielded some active sites of free amino group in WPI. Finally, non-covalent polymerization of dextran at higher reaction temperature for a certain reaction time decreased the accessible active sites of reducing carbonyl groups and its molecular mobility and diffusivity. Similar phenomena were reported in previous studies [27,28,29].

### 3.2. SDS-PAGE Analysis

**Figure 1:**
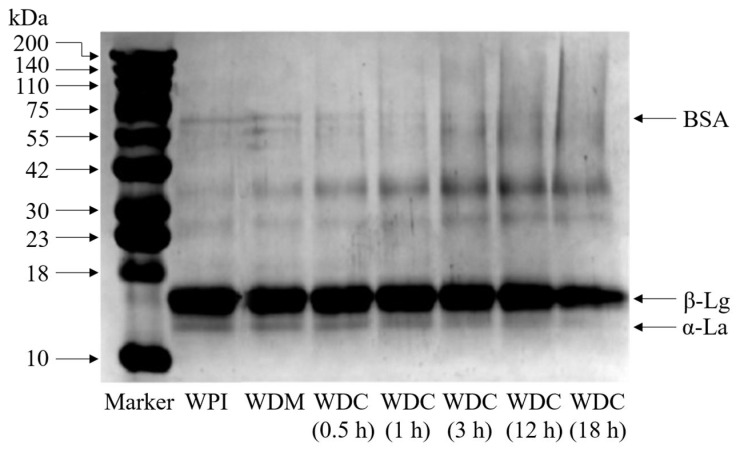
SDS–PAGE of WPI, WDM, and WDCs (0.5–18 h). Marker: molecular weight standard, WDM: WPI–dextran mixture, WDCs: WPI–dextran conjugates, obtained according to the following reaction conditions: 90% ethanol (*v*/*v*), a weight ratio of WPI to dextran 1:3 (*w*/*w*), a ratio of solid to liquid of 10% (*w*/*v*), temperature 70 °C, time (0.5, 1, 3, 12, and 18 h), recorded as WDC (0.5 h), WDC (1 h), WDC (3 h), WDC (12 h), and WDC (18 h), respectively.

Dimers or large-molecular-weight polymers of protein due to disulfide formation can completely disappear under reducing electrophoresis. When the molecular weight of the protein subunit is smaller, the mobility of the corresponding molecules is faster in SDS-PAGE [28]. To further confirm whether covalent WPI–dextran conjugates are formed or not during the Maillard reaction process, SDS-PAGE patterns were performed in the gel made up of a 12% separating gel and a 5% stacking gel. Figure 1 displays the electrophoretic characteristics of WPI, WDM, and WDCs. WPI exhibited three main bands from bottom to top at about 13.8, 18.0, and 66.0 kDa, which were attributed to α-La, β-Lg, and BSA, respectively [28].

As shown in Figure 1, WPI and WDM had identical electrophoretic bands and band intensities, demonstrating that the composition and molecular weight distribution of WPI in WDM were not changed due to the presence of non-covalent dextran in WDM. This indicated that no covalent conjugates occurred between WPI and dextran in WDM without heat treatment. Dextran in WDM could not migrate into the separating gel because dextran is a neutral molecule to the weak non-covalent interactions between dextran and WPI in SDS-PAGE [30]. Compared with WPI and WDM, the intensity of α-La, β-Lg, and BSA bands in WDCs diminished gradually with an increase in reaction time. However, new diffuse bands continuously appeared near the top of separating gel, which obviously indicated that covalent conjugates with high-molecular-weight compositions were formed between WPI and dextran. The molecular weight distribution of WDCs was broader than those of WPI and WDM, which resulted from WPI containing different molecular sizes of various proteins with an individual protein molecule covalently bonded to varying numbers of dextran. Most of β-Lg bands in WDCs remained, while α-La and BSA bands tended to disappear, indicating that the glycosylated reaction of β-Lg with dextran might not be easier than that of α-La and BSA with dextran. Previous research also reported that the loss of free NH_2_ groups per mol of reactant proteins after covalent grafting of dextran was in the order BSA > α-La > β-Lg, which was determined using the trinitrobenzenesulphonic acid (TNBS) method [4]. These phenomena were attributed to the type of protein and the number of its reactive amino groups susceptible to the Maillard reaction. For example, β-Lg, α-La, and BSA account for around 50%, 15%, and 5% of the total whey proteins and contain 162, 123, and 582 amino acid residues and comprise 16, 13, and 59 potentially reactive amino for being glycosylated, respectively [4,31]. β-Lg generally exists as a dimer of two identical subunits, and each monomer consists of two disulfide bonds and one sulfhydryl group [31].

### 3.3. FTIR Spectroscopy Analysis

The absorption band at a particular wavenumber corresponds to a vibration frequency of the specific chemical bond in an FTIR spectrum. Moreover, the intensity of absorption is proportional to the amount of absorbing substance in a sample [12]. A broad band located in the spectral range from 3000 cm^−1^ to 3600 cm^−1^ is attributed to the stretching vibration of free and bound O-H, N-H, and =C-H groups according to the previous findings [32]. The band peak in the spectral range from 2850 cm^−1^ to 2980 cm^−1^ is assigned to the stretching vibration of C-H according to a previous study [32]. Although the mid-infrared spectra of protein exhibit nine characteristic absorption bands, including amide A, B, and I-VII, the Amide I-III are most commonly used in infrared protein research [33]. The most sensitive spectral region to the protein structural changes is the Amide I band at 1600–1700 cm^−1^, which arises from the stretching vibration of C=O (70–85%) and C-N groups (20%) [32]. The absorption of the Amide II band is located in the range of 1480–1575 cm^−1^, which is governed by in-plane bending vibration of N-H (40–60%) and stretching vibration of C-N (18–40%) [33]. Although the signal intensity of the Amide III band is only one-tenth to one-fifth that of Amide I band, there is no interference of water molecules in the Amide III region [34]. The Amide III absorption in the 1200–1350 cm^−1^ region is attributed to the stretching vibration of C-N, in-plane bending vibration of N-H, and weak stretching vibration of C-C [32].

**Figure 2:**
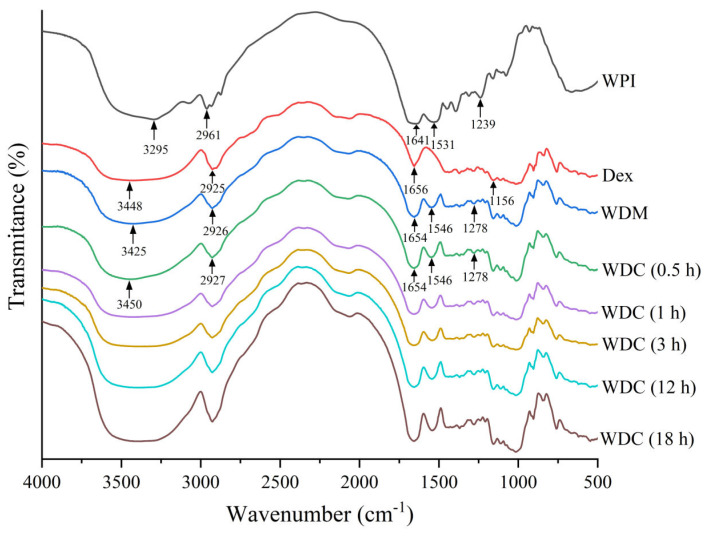
FTIR spectra of WPI, Dex, WDM, and WDCs (0.5–18 h). WDM: WPI–dextran mixture, Dex: dextran, WDCs: WPI–dextran conjugates, as in Figure 1. All spectra were plotted on the same vertical scale with an offset for clarity.

The FTIR spectra of WPI, dextran, WDM, and WDCs are shown in Figure 2. The absorption peaks of WPI in the Amide I, II, and III regions were 1641 cm^−1^, 1531 cm^−1^, and 1239 cm^−1^, respectively. After glycosylation, all absorption peaks of WDCs in the Amide I, II, and III regions showed a blueshift to 1654 cm^−1^, 1546 cm^−1^, and 1278 cm^−1^, respectively. The intensities of WDCs in the Amide I-III regions gradually decreased with an increase in reaction time from 0.5 h to 3 h and were weaker than those of WDM. These results indicated that changes in the structure and conformation of WPI occurred after glycosylation due to a decrease in the amount of N-H and C-N groups when they formed the intermediate Schiff base (C=N) following the carbonyl-amine (Maillard) reaction. Similar results were reported in previous studies [35,36]. However, the intensity of WDCs in the Amide I-III regions gradually increased with further extension of the reaction time to 12 h–18 h, which may be attributed to the production of Amadori rearrangement compounds (C=O) from Schiff base products. These results are consistent with a previous report [37]. The infrared spectra of dextran showed the stretching vibrations of C-C and C-O and bending vibration of C-H in the range of 1180–953 cm^−1^, where the protein absorption was weak. The vibrations are commonly referred to as the “saccharide” bands [38]. The intensity of WPI alone in the “saccharide” band range was lower than both those of WDM and WDCs. Similar results have been reported in a previous study [39]. The band peaks of all WDCs at 1014 cm^−1^ were higher than those of WPI due to the stretching vibrations of newly formed C-N covalent bonds via the carbonyl-amine reaction. This finding was consistent with a previous study [36]. These results of FTIR spectra indicated that the formation of WDCs was verified and both glycosylation and DG significantly influenced the molecular structure and conformation of WPI.

### 3.4. UV-Vis Absorption Spectroscopy Analysis

**Figure 3:**
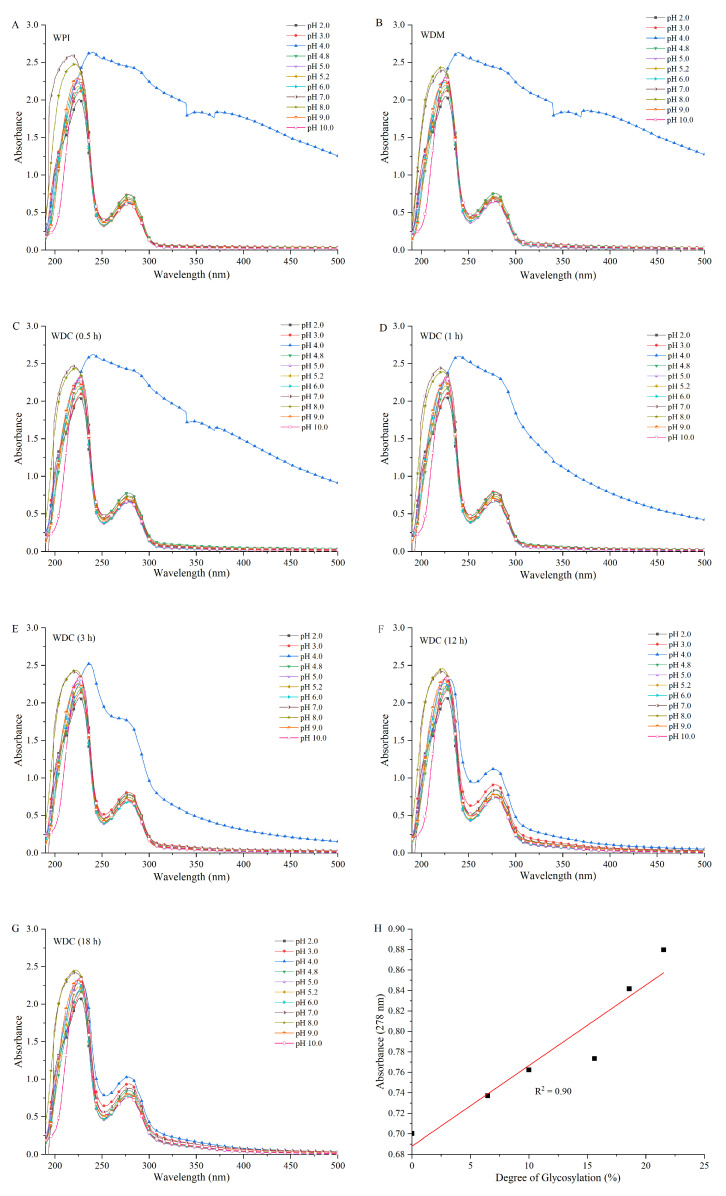
UV-Vis spectra of WPI (**A**), WDM (**B**), and WDCs (0.5–18 h) (**C**–**G**) diluted in different pH buffer solutions from 2.0 to 10.0 in the wavelength range of 190–500 nm and absorbance of WDCs at 278 nm versus their degrees of glycosylation in pH 7.0 buffer solution (**H**). The line represents the result of linear fitting in subgraph (**H**). WDM: WPI–dextran mixture, WDCs: WPI–dextran conjugates, as in Figure 1.

The absorption energy of UV-Vis light is equivalent to the electronic transition energy difference of covalently unsaturated compounds at UV-Vis light excitation [13]. UV-Vis absorption spectra of WPI, WDM, and WDCs are shown in Figure 3. The variation of the protonation and deprotonation states of WPI, WDM, and WDCs dissolved in different pH buffer solutions would influence the electronic structure of their molecules, and thus affect their characteristic absorption wavelength and intensity. WPI, WDM, and WDCs (0.5 h, 1 h, 3 h) had abnormal UV-Vis spectra at pH 4.0, which was due to a change in the protein isoelectric point from pH 4.8–5.2 to approximately pH 4.0 under 0.01 mol/L buffer solution of disodium hydrogen phosphate-citric acid. The turbidity of these samples in pH 4.0 buffer solution increased such that reliable signal-to-noise ratio of UV-Vis spectra could not be obtained. Similar results regarding the effect of ionic strength on the isoelectric point of proteins were reported in previous studies [40,41]. WDCs (12 h, 18 h) with higher DGs ((18.57 ± 0.31)%, (21.51 ± 0.27)%) had normal UV-Vis spectra at pH 4.0, which was attributed to an increase in solubility of WPI after covalent grafting of dextran. WDC with DGs beyond (15.68 ± 0.20)% had good solubility in our previous study [8]. WPI, WDM, and WDCs in buffer solutions with pH range from 2.0 to 10.0 except for pH 4.0 exhibited two absorption bands in the ranges of 200–240 nm and 260–290 nm, which corresponded to the peptide backbone and aromatic amino acid residues, respectively. These results are in agreement with previous studies [6,14,17,42]. There was no measurable absorbance at wavelengths longer than 320 nm.

The maximum absorption wavelength (λ_max_) of WPI, WDM, and WDCs in the range of 200–240 nm gradually shifted to a shorter wavelength (blueshift) when the pH values of their sample solutions were altered from pH 4.8 to pH 3.0–2.0 or pH 5.0–7.0 and subsequently shifted to a longer wavelength (redshift) beyond pH 7.0. Glycosylation could improve the stability of WPI and partly prevent the shift of the λ_max_ of WPI in different pH buffer solutions. For instance, the λ_max_ of WPI and WDC (18 h) shifted from 227 nm at pH 4.8 to 218 nm and 222 nm at pH 7.0, respectively. The absorption intensities of WPI, WDM, and WDCs in the range of 200–240 nm gradually decreased and exhibited hypochromism when pH of their sample solutions was altered from pH 5.0 to pH 4.8 and pH 3.0 to 2.0, gradually increased and exhibited hyperchromism from pH 5.0 to pH 5.2–7.0, and eventually decreased and exhibited hypochromism beyond pH 7.0. Similar results regarding the effect of pH on the λ_max_ and intensity of UV-Vis spectra were reported in previous studies [43,44].

The λ_max_ of WPI, WDM, and WDCs at around 278 nm in the range of 260–290 nm showed no significant difference under different buffer solutions in the range of pH 2.0–10.0 except for pH 4.0. An analogous trend was observed in the absorption intensities of WPI, WDM, and WDCs in the range between 200–240 nm and 260–290 nm. For the UV-Vis spectra in the range of 260–290 nm, the absorption intensities of WDCs in different pH buffer solutions were higher than those of WPI and WDM, and gradually increased with an increase in the DGs. Apparent hyperchromism was also exhibited, which was attributed to the formation of Maillard reaction products, such as the intermediate Schiff bases, and the increase in exposed aromatic amino acid residues of glycosylated WPI following extension of molecular chains. These results indicated that pH and glycosylation could change the conformation of WPI and glycosylation could also induce the structural change of WPI. These results are in agreement with previous studies [6,42]. The UV-Vis absorption intensities of WDC samples in the range of 260–290 nm were highly and positively correlated with their DGs. The linear regression equation of UV-Vis absorption intensity versus DG of WDC was as follows: I_abs_ = 0.008DG + 0.688 (R^2^ = 0.90), where I_abs_ and DG represent the absorbance at 278 nm and the degree of glycosylation of WDC, respectively. R^2^ is the correlation coefficient of the fitting curve. Therefore, UV-Vis absorption spectroscopy appears to be a promising technique for quantitatively monitoring the degree of glycosylation in a rapid, sensitive, low-cost, and practical way without any chemical reagents. These results suggested that UV-Vis absorption intensity of proteins after glycosylation in the range of 260–290 nm would increase with an increase in the degree of glycosylation of conjugates and indirectly indicate the enhancement in solubility and consequently improve other functional properties of glycosylated proteins based on their solubility, such as stability, emulsifying, and foaming properties.

The changes in the λ_max_ of WPI, WDM, and WDCs in the range of 200–240 nm and in their absorption intensities in the range of 200–240 nm and 260–290 nm were related to the protonation or deprotonation states of carbonyl groups and free amino groups in native and glycosylated WPI, such as the protonation of most carbonyl groups and completely free amino groups at lower pH values (2.0–3.0), deprotonation of carbonyl groups and protonation of completely free amino groups in the pH range from 4.8 to 7.0, and deprotonation of both carbonyl groups and most free amino groups beyond pH 7.0. In addition, the λ_max_ and intensity of UV-Vis spectra of WPI, WDM, and WDCs beyond the isoelectric point showed opposing changing trends in the same band range.

### 3.5. Intrinsic Fluorescence Emission Spectroscopy Analysis

The improvement in sensitivity of fluorescence spectroscopic technique is due to usage of two wavelength parameters of excitation and emission compared with other spectroscopic techniques based only on absorption [45]. Fluorescence spectroscopy is a rapid, sensitive, and highly specific method for characterizing the changes of local microenvironmental polarity around the fluorescent groups (fluorophores) in protein molecules, and thus is usually used to examine the structural and conformational changes of proteins, which are measured by the variation of the λ_max_ and intensity of a protein fluorescence spectrum [16]. The λ_max_ of a protein-intrinsic fluorescence emission spectrum shifts to a shorter wavelength, which is called a blueshift of λ_max_, suggesting that the fluorophores in a protein are in more hydrophobic interior and protein molecules are aggregated with each other. On the contrary, the shift of λ_max_ to a longer wavelength, which is called a redshift of λ_max_, is an indication that the fluorophores are exposed to the hydrophilic solvent and protein molecules are unfolded [46]. The intensity of a protein intrinsic fluorescence emission spectrum reflects the average exposure of protein fluorophores to the aqueous phase [18]. The increase in protein fluorescence intensity is related to dissociation of protein aggregates and therefore, more exposure of fluorophores initially inside the molecules to the surface [47,48,49]. However, the decrease in protein fluorescence intensity is correlated with quenching under diverse conditions [50]. Although proteins have three aromatic fluorophores, including Trp, Tyr, and Phe residues, the intrinsic fluorescence mainly originates from Trp and Tyr residues due to a very low quantum yield of Phe residues [18]. The total intrinsic fluorescence emission spectrum of both Tyr and Trp residues in the hydrophobic pockets of globular proteins can be obtained at an excitation wavelength of 280 nm [51].

**Figure 4:**
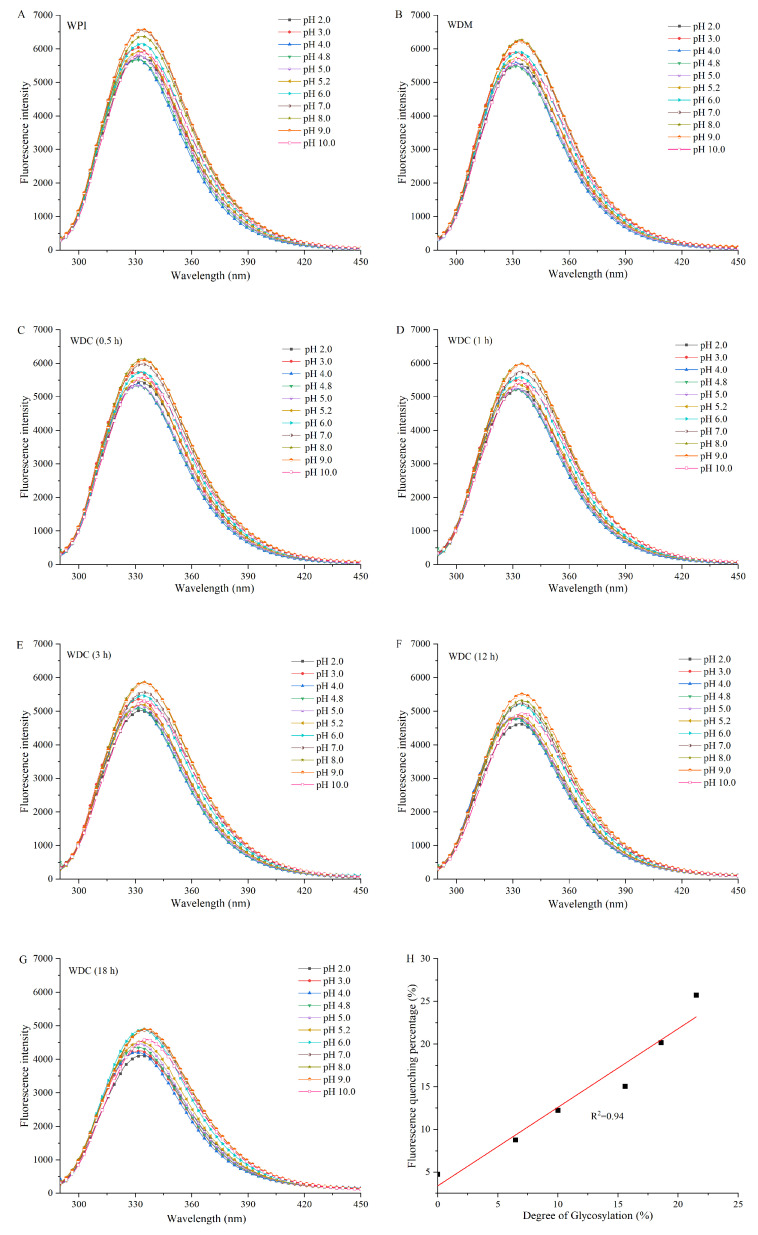
Intrinsic fluorescence emission spectra of WPI (**A**), WDM (**B**), and WDCs (0.5–18 h) (**C**–**G**) diluted in different pH buffer solutions from 2.0 to 10.0 at 280 nm excitation wavelength and fluorescence quenching percentage of WDCs at 280 nm excitation wavelength versus their degrees of glycosylation in pH 7.0 buffer solution (**H**). The line represents the result of linear fitting in subgraph (**H**). WDM: WPI–dextran mixture, WDCs: WPI–dextran conjugates, as in Figure 1.

Intrinsic fluorescence emission spectra of WPI, WDM, and WDCs are shown in Figure 4. β-Lg and α-La, two main components of WPI, contain 4 and 4 Tyr residues and 2 and 4 Trp residues per molecule, respectively [52]. Tyr and Trp residues in WPI, WDM, and WDCs exhibited distinct and typical intrinsic fluorescence emission spectra at the excitation wavelength of 280 nm. The λ_max_ of WPI was 330.6 nm at pH 4.0. The λ_max_ of WPI was a slight redshift from pH 4.0 to pH 3.0–2.0. An obvious redshift from pH 4.0 to pH 4.8–10.0 indicated that Tyr and Trp residues in WPI were situated in a more polar and hydrophilic microenvironment. The λ_max_ of WPI in near neutral and alkaline buffer solutions (pH 6.0–10.0) had a redshift compared with that of WPI in acidic buffer solutions (pH 2.0–5.2), which may be attributed to the fact that the molecular structure of WPI was more stretched in the high pH range, therefore contributing to a more polar and hydrophilic microenvironment around Tyr and Trp residues. Similar findings were reported in previous studies [46,53]. Previous research reported that Trp residues were buried in a “non-polar” environment if λ_max_ of Trp fluorescence was <330 nm, and Trp residues were located in a “polar” environment if λ_max_ was >330 nm, indicating that Trp residues were exposed to the solvent [48]. Compared with the intensity of WPI at pH 4.0, the intensity of WPI increased in the pH range from 2.0 to 3.0. There was also a gradual increase in intensity of WPI in the pH range from 4.8 to 10.0 except for fluctuating increases at pH 8.0 and pH 10.0. The UV-Vis spectrum of WPI at pH 4.0 also confirmed the compact aggregate state at pH 4.0, which might be related to the strong intramolecular interactions such as hydrogen bond formation and hydrophobic interactions. Therefore, Trp residues located inside a more non-polar and hydrophobic microenvironment of the aggregate were then excited to lower intensity of fluorescence at pH 4.0. WPI molecules converted from a compact and tight structure to a loose and expansive state when pH values were altered from 4.0 to 3.0–2.0 and 4.8–10.0. Accordingly, Trp residues exposed to a more polar and hydrophilic microenvironment exhibited higher intensity of fluorescence in the pH ranges of 2.0–3.0 and 4.8–10.0. Additionally, partial unfolding of WPI molecules at a pH away from 4.0 might decrease the internal self-quenching of fluorescence. However, the intensity of WPI at pH 10.0 was lower than that of WPI at pH 9.0, which was attributed to quenching of fluorescence by deprotonation of Tyr phenolic groups. Similar results were reported in a previous study [50]. The λ_max_ and intensity of fluorescence spectra of WPI, WDM, and WDCs exhibited similar trends as described above. The λ_max_ of fluorescence spectra of WPI, WDM, and WDC (18 h) shifted from 330.6 nm, 330.0 nm, and 329.6 nm at pH 4.0 to 332.6 nm, 333.0 nm, and 333.8 nm at pH 2.0, and 333.6 nm, 334.4 nm, and 336.0 nm at pH 10.0, respectively.

Dextran in either mixture or conjugate form could cause conformational changes in WPI and increase the polarity and decrease the hydrophobicity around the Trp and Tyr residues, which could further induce more exposure of Trp and Tyr residues with increased DGs. These findings suggested that WDC molecules had a good solubility and did not tend to aggregate with each other by hydrophobic interactions. Similar results were reported in previous studies [54,55]. The intensity of fluorescence spectra of WPI, WDM, and WDCs with increased DGs significantly decreased in the same order at similar pH values, indicating that fluorescence quenching of covalent grafting of dextran in conjugates on Trp and Tyr residues was stronger than that of noncovalent grafting of dextran in WDM and fluorescence quenching of covalent grafting of dextran increased with an increase in the DGs. Fluorescence quenching percentage was calculated by using the fluorescence intensity difference between WPI and WDC divided by fluorescence intensity of WPI. The linear regression equation of fluorescence quenching percentage versus DG of WDC was as follows: I_int_ = 0.920DG + 3.397 (R^2^ = 0.94), where I_int_ and DG represent the intensity at the excitation wavelength of 280 nm and the degree of glycosylation of WDC, respectively. R^2^ is the correlation coefficient of the fitting curve. Therefore, fluorescence quenching of covalent grafting of dextran on WDCs was highly and positively correlated with their DGs. In addition, the fluorescence intensity of WPI, WDM, and WDCs in near neutral and alkaline buffer (pH 6.0–9.0) was higher than when samples were in acidic buffer (pH 2.0–5.2) solutions, suggesting that WPI, WDM, and WDCs in former solutions were exposed to a greater number of OH groups and ionization of a carboxylic acid group and Trp and Tyr residues in protein molecules. These results are in agreement with previous findings [47,49].

### 3.6. Synchronous Fluorescence Spectroscopy Analysis

Total intrinsic fluorescence of Trp and Tyr residues in protein molecules can be detected at the excitation wavelength of 280 nm. Although the intrinsic fluorescence of Trp residues in protein molecules can be measured at the excitation wavelength of 295 nm, the intrinsic fluorescence of Tyr residues cannot be obtained at a specific excitation wavelength. Synchronous fluorescence spectroscopy can collect characteristic fluorescence of separate Tyr and Trp residues at a fixed wavelength difference (Δλ) of 15 nm and 60 nm, respectively, which is the emission wavelength minus excitation wavelength [45]. Synchronous fluorescence spectroscopy exhibits several notable advantages, including a narrowing of spectral band, an enhancement in selectivity through spectral simplification, a decrease in measurement time in multicomponent analysis, and a reduction in or avoidance of different interference effects [45,56]. Similar to intrinsic fluorescence emission spectroscopy, synchronous fluorescence spectroscopy is frequently used to evaluate the changes in polarity and hydrophobicity in the vicinity of protein fluorophores based on the shift in the position of λ_max_ [52].

**Figure 5:**
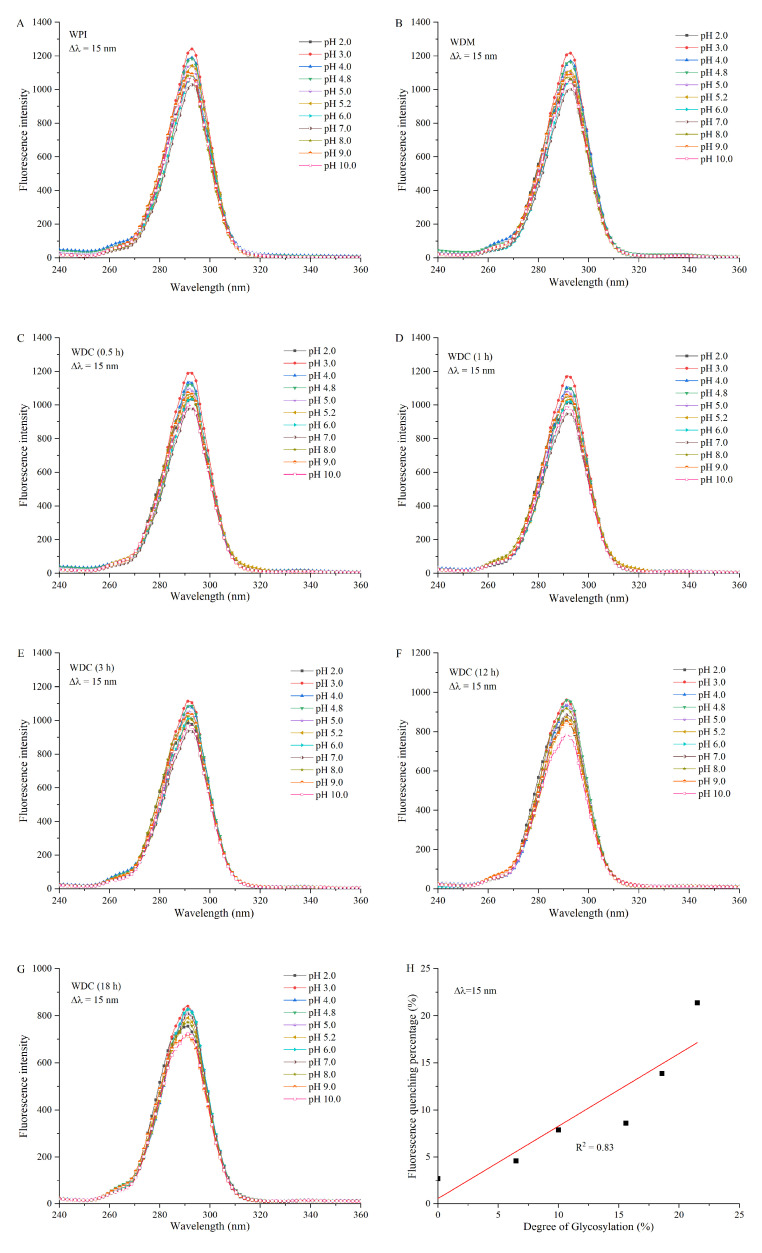
Synchronous fluorescence spectra of WPI (**A**), WDM (**B**), and WDCs (0.5–18 h) (**C**–**G**) diluted in different pH buffer solutions from 2.0 to 10.0 at a fixed emission-excitation wavelength difference (Δλ) of 15 nm and fluorescence quenching percentage of WDCs at Δλ = 15 nm versus their degrees of glycosylation in pH 7.0 buffer solution (**H**). The line in subgraph H represents the result of linear fitting. WDM: WPI–dextran mixture, WDCs: WPI–dextran conjugates, as in Figure 1.

Synchronous fluorescence spectra of Tyr residues in WPI, WDM, and WDCs are shown in Figure 5. They were significantly different from intrinsic fluorescence emission spectra of Tyr and Trp residues in the three samples. The λ_max_ of Tyr residues in WPI and WDM was around 292.6 nm in the entire experimental pH range of 2.0–10.0, indicating that the hydrophobic microenvironment around Tyr residues in WPI and WDM was not significantly altered under different pH conditions. Compared with the intensity of Tyr residues in WPI and WDM at pH 4.0, the intensity of Tyr residues in WPI and WDM first increased and then decreased in the pH range from 3.0 to 2.0 and gradually decreased in the pH range from 4.8 to 7.0. Subsequently the intensity increased and then decreased in the pH range from 8.0 to 10.0. The increase in intensity at pH 3.0 and pH 8.0–9.0 may be attributed to the unfolding of proteins and therefore, more exposure of Tyr residues on the surface. The decrease in intensity at pH 2.0 may be due to the quenching of more hydrogen ions, protonated amino groups, protonated histidine residues, and neutralization of COO- groups in aspartic and glutamic acid residues around Tyr residues. Similar findings were reported in previous studies [18,50]. The decrease in intensity in the pH ranges from 4.8 to 7.0 resulted from the renaturation and subsequent rearrangement of WPI and self-quenching of Tyr residues. The decrease in intensity at pH 10.0 was due to the quenching of fluorescence via deprotonation of Tyr phenolic groups. Although synchronous fluorescence spectra of Tyr residues in WPI, WDM, and WDCs had a similar trend, the λ_max_ and intensity of Tyr residues in WDCs with increased DGs exhibited a slight blueshift and a decrease compared with those in WPI and WDM, respectively, an indication that covalent grafting of dextran changed the structure and conformation of WPI, resulting in burial of Tyr residues in a more non-polar and hydrophobic microenvironment and in quenching of fluorescence. The linear regression equation of fluorescence quenching percentage versus DG of WDC was as follows: I_int_ = 0.770DG + 0.592 (R^2^ = 0.83), where I_int_ and DG represent the intensity at Δλ = 15 nm and the degree of glycosylation of WDC, respectively. R^2^ is the correlation coefficient of the fitting curve. Therefore, fluorescence quenching of covalent grafting of dextran on Tyr residues in WDCs was positively correlated with their DGs.

**Figure 6:**
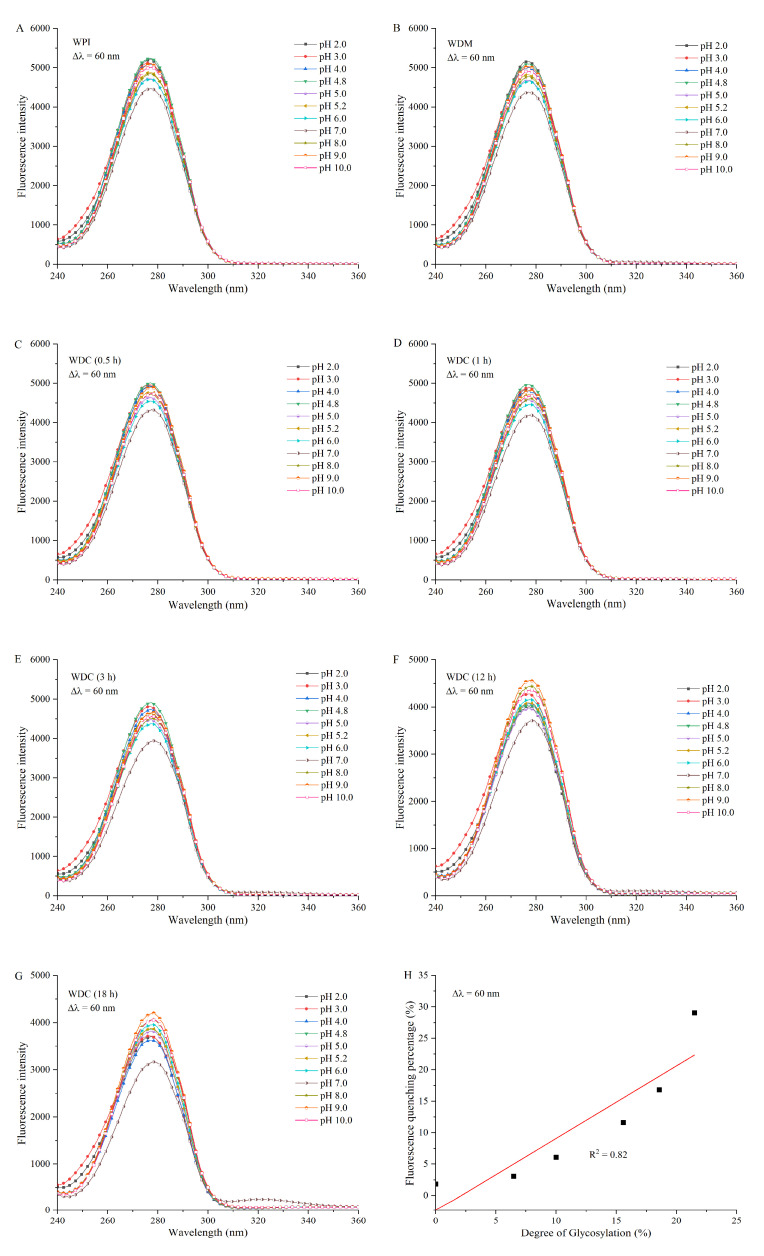
Synchronous fluorescence spectra of WPI (**A**), WDM (**B**), and WDCs (0.5–18 h) (**C**–**G**) diluted in different pH buffer solutions from 2.0 to 10.0 at a fixed emission-excitation wavelength difference (Δλ) of 60 nm, and fluorescence quenching percentage of WDCs at Δλ = 60 nm versus their degrees of glycosylation in pH 7.0 buffer solution (**H**). The line in subgraph H represents the result of linear fitting. WDM: WPI–dextran mixture, WDCs: WPI–dextran conjugates, as in Figure 1.

Synchronous fluorescence spectra of Trp residues in WPI, WDM, and WDCs are shown in Figure 6. They were significantly different from those of Tyr residues in the three samples. The peak shape and intensity of the former were broader and higher than those of the latter. The intensities of synchronous fluorescence spectra of Trp residues in WPI, WDM, and WDCs were closest to those of total intrinsic fluorescence emission spectra of both Tyr and Trp residues in the corresponding samples, respectively, indicating that the fluorescence of the proteins was predominantly from Trp residues. The λ_max_ of Trp residues in WPI and WDM was around 276.8 nm in the entire experimental pH range of 2.0–10.0, indicating that the microenvironmental hydrophobicity around Trp residues in both was not significantly altered under different pH conditions. The order of fluorescence intensity of Trp residues in WPI and WDM was as follows: low pH (2.0–4.8) > high pH (9.0–10.0) > around neutral pH (5.0–8.0). The higher intensity at low and high pH ranges may be attributed to the unfolding of proteins and therefore, more exposure of Trp residues on the surface. The lower intensity near neutral pH range may be due to the renaturation and subsequent rearrangement of WPI and self-quenching of Trp residues.

Compared with the synchronous fluorescence spectrum of Trp residues in WPI, the λ_max_ of Trp residues in WDCs increased with an increase in the DGs until a redshift occurred. The redshift of λ_max_ of Trp residues in WDCs with increased DGs in alkaline solutions was greater than that of the same residues in acidic solutions. These results indicated that covalent grafting of dextran changed the structure and conformation of WPI and then induced exposure of Trp residues to a more polar and hydrophilic microenvironment with an increase in the DGs and pH. These findings suggested that WDC molecules had a good solubility and did not form aggregates by hydrophobic interactions. The intensity of Trp residues in WDCs with increased DGs gradually decreased in the pH range of 2.0–10.0, suggesting that more covalent grafting of dextran quenched the fluorescence of Trp residues. The reversal in fluorescence intensity between low pH (2.0–4.8) and high pH (9.0–10.0) indicated that the fluorescence quenching of Trp residues in WDCs in acidic solutions, caused by hydrogen ions, protonated amino group, protonated histidine residues, and neutralization of COO- groups in aspartic and glutamic acid residues around Trp residues as well as covalent grafting of dextran, was stronger than in alkaline solutions, where there was only covalent grafting of dextran. Similar findings were reported in previous studies [57,58]. The intensity of Trp residues in all WPI, WDM, and WDCs at pH 7.0 was lowest in the entire experimental pH range from 2.0 to 10.0, demonstrating that the renaturation and subsequent rearrangement at pH 7.0 induced the strongest fluorescence self-quenching of Trp residues.

The linear regression equation of fluorescence quenching percentage versus DG of WDC was as follows: I_int_ = 1.155DG − 2.468 (R^2^ = 0.82), where I_int_ and DG represent the intensity at Δλ = 60 nm and the degree of glycosylation of WDC, respectively. R^2^ is the correlation coefficient of the fitting curve. Therefore, fluorescence quenching of covalent grafting of dextran on Trp residues in WDCs was positively correlated with their DGs. The Maillard reaction can affect food quality by altering color, aroma, flavor, stability, and nutritional value. Fluorescence spectroscopy have been used to rapidly detect the Maillard reaction. Fluorescence quenching of glycosylate proteins would increase with an increase in the degree of glycosylation, suggesting that the structure and conformation of proteins could be changed after the Maillard reaction, thereby improving their functional properties such as stability, emulsifying, and foaming. Excessive Maillard reaction would compromise the quality and shelf life of food [59]. Therefore, the extent of fluorescence quenching of glycosylate proteins should be controlled to obtain specific functional properties of proteins or used to evaluate product quality, including quality and safety of food products [60,61].

## 4. Conclusions

The functional properties of proteins are closely related to their structure and conformation. Different spectroscopies can capture characteristic spectral signals of different functional groups of proteins and have different sensitivities. The successful preparation of WDCs through the Maillard reaction was confirmed by DG, SDS-PAGE, and FTIR spectroscopy. A blueshift of FTIR peaks of WPI after glycosylation occurred in the Amide I, II, and III regions, accompanied by a decrease in intensity. UV-Vis absorption and fluorescence spectroscopies indicated that the changes in the λ_max_ and intensity of WPI were closely related to the protonation states of carbonyl groups and the free amino groups, and the degree of glycosylation. Glycosylation could prevent the λ_max_ shift of UV-Vis spectrum of WPI in the range of 200–240 nm. There was no significant difference in the λ_max_ of UV-Vis spectrum of WPI, WDM, and WDCs in different buffer solutions within the pH range of 2.0–10.0. The absorption intensities of WDCs in different pH buffer solutions were higher than those of WPI and WDM, and gradually increased with an increase in the degree of glycosylation, which was attributed to the formation of Maillard reaction products such as the intermediate Schiff bases and an increase in the exposed aromatic amino acid residues in WPI after glycosylation. Furthermore, the UV-Vis absorption intensities of WDCs at λ_max_ around 278 nm were highly and positively correlated with their DGs. The λ_max_ of total intrinsic fluorescence spectra of Tyr and Trp residues in WDCs with an increase in the DGs had an obvious redshift and the redshift of their λ_max_ near neutral and alkaline buffer solutions (pH 6.0–10.0) was more significant than in acidic buffer solutions (pH 2.0–5.2). The intensities of synchronous fluorescence spectra of individual Trp residues in WPI, WDM, and WDCs were closest to those of total intrinsic fluorescence emission spectra of Tyr and Trp residues in those samples, respectively, indicating that the fluorescence of proteins was predominantly from Trp residues. The fluorescence quenching of covalent grafting of dextran on WDCs with an increase in the DGs gradually increased regardless of whether it was observed in total intrinsic fluorescence spectra of Tyr and Trp residues and synchronous fluorescence spectra of individual Tyr or Trp residues. The λ_max_ shift of synchronous fluorescence spectra of individual Tyr or Trp residues in WDCs with an increase in the DGs gradually increased and the former and latter had a blueshift and redshift, respectively. Synchronous fluorescence spectra of WDCs suggested that covalent grafting of dextran in WDCs changed the structure and conformation of WPI, where burial of Tyr residues occurred in a more non-polar and hydrophobic microenvironment with an increase in the DGs. On the contrary, the covalent grafting of dextran in WDCs induced the exposure of Trp residues to a more polar and hydrophilic microenvironment with an increase in the DGs and pH, suggesting that WDC molecules had a good solubility and did not tend to aggregate with each other through hydrophobic interactions. Consequently, this work will be beneficial for understanding the structural and conformational changes of proteins by measuring the microenvironment around Tyr and/or Trp residues in proteins using UV-Vis absorption spectroscopy and synchronous fluorescence spectroscopy. It will also provide a promising alternative for quantitatively monitoring the DG in a rapid and practical way without any chemical reagents, using UV-Vis absorption spectroscopy in the range of 260–290 nm.

The findings suggested that UV-Vis absorption intensity and fluorescence quenching would increase with an increase in the degree of glycosylation of proteins and thus could be simple, quick, low-cost potential alternative methods to detect the degree of glycosylation. Although the Maillard reaction could endow with good color, aroma, and flavor of food products and improve the functional properties of protein such as stability, foaming, and emulsifying, excessive glycosylation would produce some harmful substances and destroy quality and safety of food. Compared with fluorescence spectroscopy, UV-Vis absorption spectroscopy is a simpler, sensitive, and low-cost method for determining the degree of glycosylation. Therefore, the UV-Vis absorption spectroscopy would be beneficial for precisely controlling the Maillard reaction process during food processing, thereby obtaining good quality and safety in food products while avoiding the generation of harmful ingredients.

## Figures and Tables

**Table 1:** Degree of glycosylation of WDCs (0.5–18 h). WDCs: WPI–dextran conjugates, prepared according to the following reaction conditions: 90% ethanol (*v*/*v*), a weight ratio of WPI to dextran 1:3 (*w*/*w*), a ratio of solid to liquid of 10% (*w*/*v*), temperature 70 °C, time (0.5, 1, 3, 12, and 18 h), respectively. Different lowercase letters (a–e) indicate significant differences (*p* < 0.05).

Reaction Time (h)	Degree of Glycosylation (%)
0.5	6.47 ± 0.31 ^e^
1	9.99 ± 0.16 ^d^
3	15.59 ± 0.16 ^c^
12	18.57 ± 0.31 ^b^
18	21.51 ± 0.28 ^a^

Data are presented as mean ± standard deviation (n = 3). Different lowercase letters indicate significant differences (*p* < 0.05).

## Data Availability

The original contributions presented in the study are included in the article. Further inquiries can be directed to the corresponding author.
